# Supplementary material for: Drosophila DAxud1 Has a Repressive Transcription Activity on Hsp70 and Other Heat Shock Genes
Source: Int J Mol Sci. 2023 Apr 19;24(8):7485. doi: 10.3390/ijms24087485 (PMC10138878; doi:10.3390/ijms24087485)
Supplement: Supplementary file 1 [file ijms-24-07485-s001.zip › Suppementary_Figures_with_caption.pdf]

Supplementary Figures

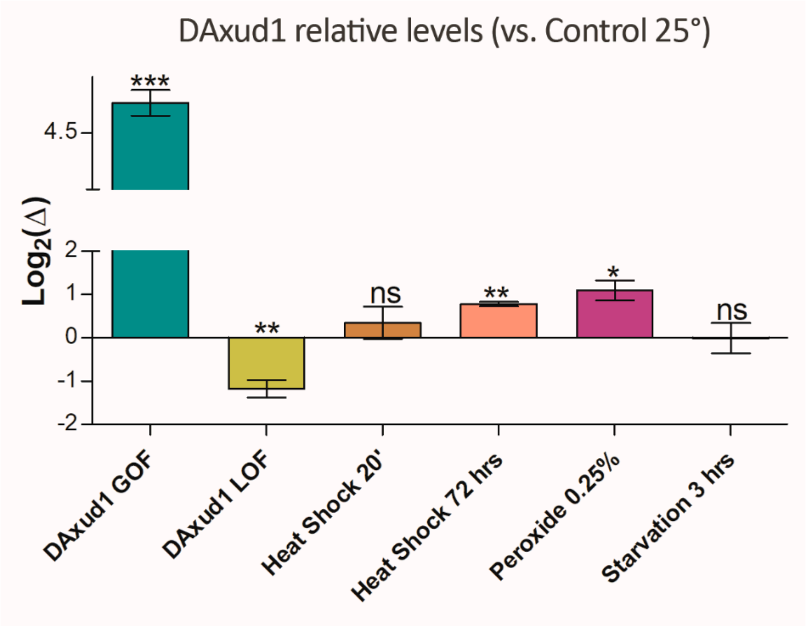

Supplementary Figure S1. DAxud1 qPCR for multiple conditions to validate genetic constructs and get exploratory information about their fluctuation with respect to control conditions (25°C, *Gal4; +/´+)* in salivary glands. DAxud1 GOF as gain of function in *nub-Gal4; UAS-DAxud1::GFP*. DAxud1 LOF from *nub-Gal4; UAS-IR DAxud1(V26479).* Heat shock 20′: acute heat shock with larvae exposed to 37°C environment for 20 minutes. Heat shock 72 hours: long exposure to heat shock at 33°C. Peroxide 0.25%: peroxide-supplemented food given to second- to third-instar larvae for 24 hours. Starvation: 3 hours in PBS 1% agarose with no nutrients. *t-test* was performed between samples using dCt (condition vs control) in triplicates, with * for $p < 0.05$, ** for $p < 0.01$ and *** for $p < 0.001$

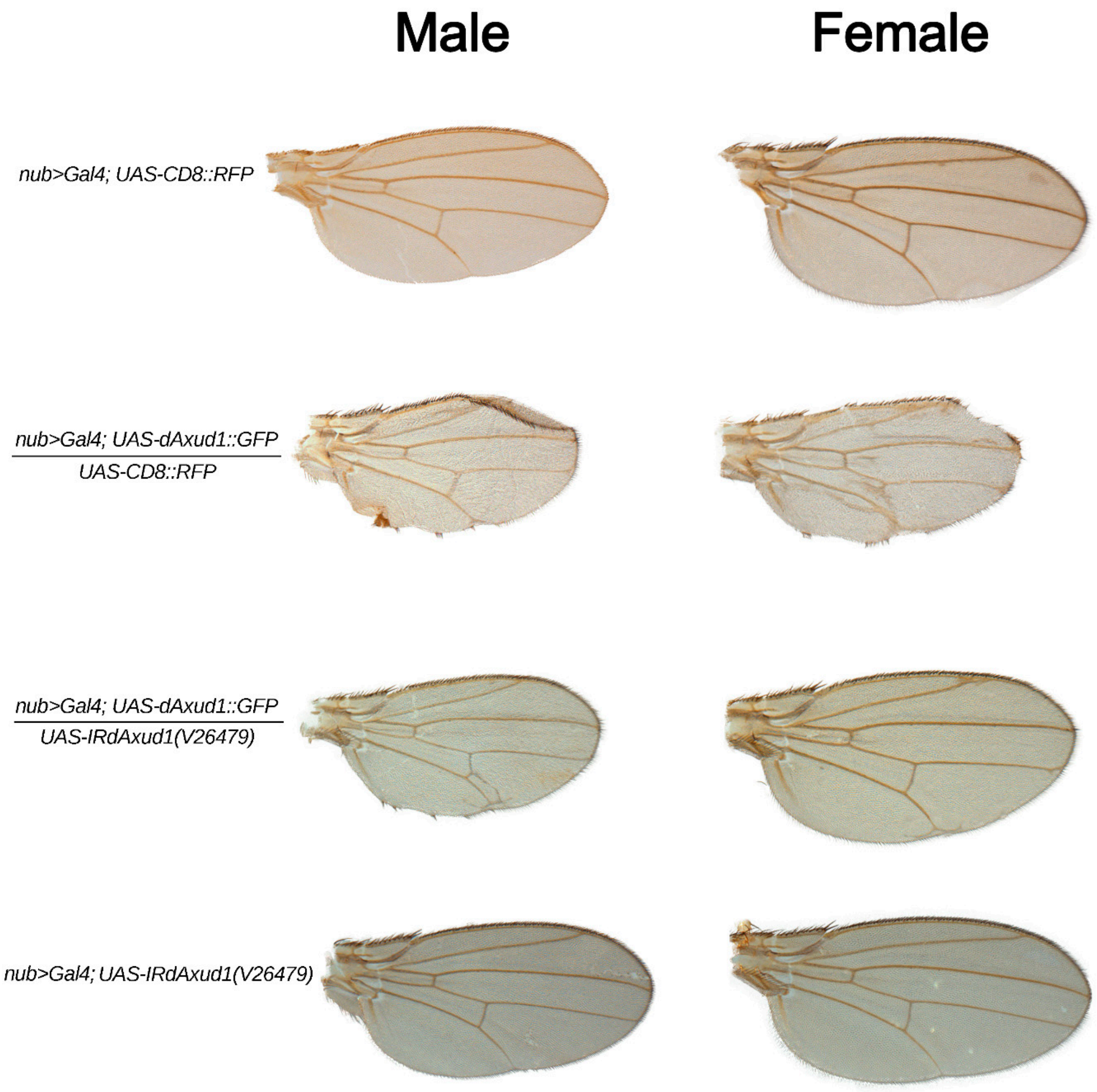

Supplementary Figure S2. Phenotype analysis of overexpression of *DAxud1* (Gain of Function, GOF) and knockdown of *dAxud1* RNAi (Loss of Function, LOF). Co-expression of *IR-dAxud1* can reverse the phenotype of overexpression of *dAxud1.* There is no evident phenotype of *dAxud1* RNA knockdown. Flies were grown at 29°C.

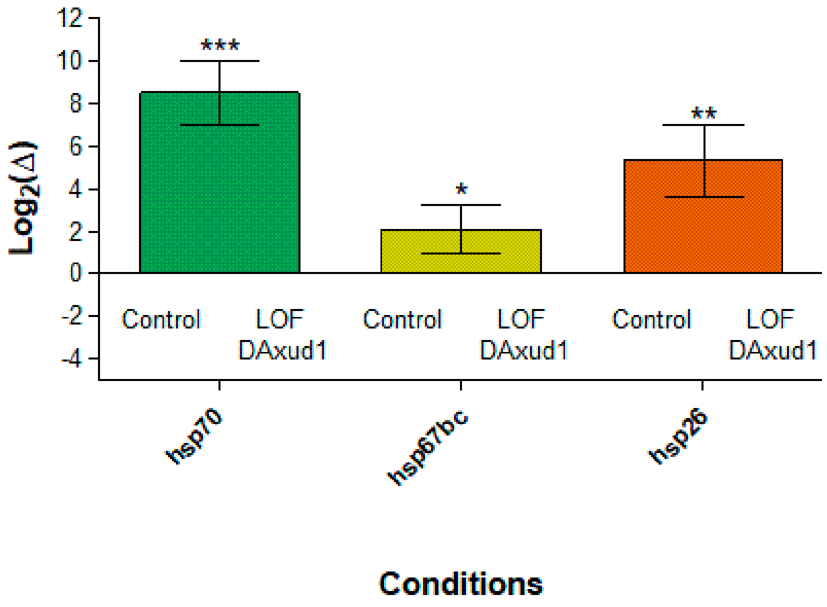

Supplementary Figure S3: qPCR for *hsp70B* paralogs, *hsp67Bc* and *hsp26* genes for test their levels in 3 day old adults at 29°C comparing control genetic background (+/+;Tub-Gal4/+) and DAxud1 Loss of function (LOF, UAS-dAxud1 RNAi V26479; Tub-Gal4/+). *t-test* was performed between samples using dCt (condition vs control) in triplicates, with * for $p < 0.05$, ** for $p < 0.01$ and *** for $p < 0.001$
